# Influence of Acrylic Acid on Kinetics of UV-Induced Cotelomerization Process and Properties of Obtained Pressure-Sensitive Adhesives

**DOI:** 10.3390/ma13245661

**Published:** 2020-12-11

**Authors:** Agnieszka Kowalczyk, Mateusz Weisbrodt, Beata Schmidt, Konrad Gziut

**Affiliations:** Department of Chemical Organic Technology and Polymeric Materials, Faculty of Chemical Technology and Engineering, West Pomeranian University of Technology in Szczecin, 70-322 Szczecin, Poland; mateusz.weisbrodt@zut.edu.pl (M.W.); beata.schmidt@zut.edu.pl (B.S.); konrad.gziut@zut.edu.pl (K.G.)

**Keywords:** pressure-sensitive adhesives, telomerization, bulk photopolymerization, polyacrylates, adhesion, adhesive joints

## Abstract

A new environmentally friendly method of photoreactive pressure-sensitive adhesives (PSAs) preparation was demonstrated. PSAs based on *n*-butyl acrylate (BA), acrylic acid (AA) and 4-acryloyloxy benxophenone (ABP) were prepared via the UV-induced cotelomerization process in the presence of a radical photoinitiator (acylphosphine oxide) and telogen (tetrabromomethane). Hydroxyterminated polybutadiene was used as a crosslinking agent. Influence of AA concentration (0–10 wt %) on kinetics of the cotelomerization process was investigated using a photodifferential scanning calorimetry method, selected physicochemical features of obtained photoreactive BA/AA/ABP cotelomers (molecular masses, polydispersity, monomers conversion and dynamic viscosity) and self-adhesive properties of obtained PSAs (adhesion, tack and cohesion) were studied, as well. It turned out that AA content is the important factor that influences monomers conversion (thereby the volatile parts content in prepolymer) and PSAs’ properties. As the acrylic acid content increases, the reaction rate increases, but the total monomers conversion and the solid content of the prepolymer decreases. Additionally, the adhesion and cohesion of PSAs were grown up, and their tackiness decreased. However, the AA content has no effect on molecular weights (Mw and Mn) and polydispersity (c.a. 1.5) of photoreactive cotelomers. The optimal AA content necessary to obtain a prepolymer with low volatile parts content and good PSA properties was determined.

## 1. Introduction

Pressure-sensitive adhesives (PSAs) are ubiquitous and exceptional materials. They are used in most consumer applications and many industrial assembly operations. PSAs are unique because they create a strong bond with the surface with only finger pressure and they do not require activation (e.g., heat or water) [1,2]. For a long time, natural rubber [poly(1,4-cis-isoprene)] was used as base material for PSAs [3]. Since 1929 it is known that alkyl acrylate ester had the characteristic tack of PSAs. However, the first formulations based on polybutyl acrylate and polyiso-butyl ether did not have the required tack and cohesive strength. Only in the 1950s, it was found that the performance of PSAs could be substantially improved by adding certain amounts of acrylic acid [4]. Today PSAs are made from a great variety of elastomers, i.e., natural, butyl, nitrile, styrene butadiene rubbers and polyurethanes, polyether, ethylene-vinyl acetate copolymers (EVA) and silicones [5]. However, polyacrylate adhesives are the most widely used [6]. Acrylic PSAs consists mainly of a “soft” monomer with a low glass transition temperature (T_g_; i.e., butyl acrylate, iso-octyl acrylate and 2-ethylhexyl acrylate) and a “hard” monomer with high T_g_ values of its homopolymer (i.e., methyl methacrylate, vinyl acetate and styrene) [7]. Nevertheless, acrylic PSAs must be chemically crosslinking in order to obtain the cohesive strength. For this purpose, polar monomers are incorporated (i.e., acrylic acid, 2-hydroxyl ethyl acrylate or *N*-vinyl pyrrolidone). However, in its simplest form, a good PSA is made from 95% of a soft monomer and 5% of acrylic acid [8]. Although the vast majority of adhesives are obtained by free radical polymerization (in the presence of the organic solvent), new methods of preparation are under development. Recently, solvent-free methods have been very popular, especially free radical bulk photopolymerization [9]. The process is initiated by the decomposition of the photoinitiator into radicals and occurs in a large volume of reagents (monomers), which are mechanical mixed during UV irradiation. The UV irradiation process is very short and lasts from a few to several dozen minutes. This is its unquestionable advantage compared to the classic radical polymerization in an organic solvent. The final product is the solution of the polymer in unreacted monomers called polymer syrup or prepolymer. An indispensable step in the preparation of PSAs by this method is the modification of the prepolymer with an additional portion of the photoinitiator and crosslinking agent and then UV irradiation of the adhesive composition, often in the presence of an inert gas or protective film. This is usually due to the high presence of unreacted monomers in the prepolymer. Nevertheless, the method is classified as proecological because it does not generate waste (recovered organic solvents or unreacted monomers) and is energy-consuming. This method was first introduced in the context of obtaining optically pure adhesives [10]. Significant is that the authors focused on testing new monomers (tetrahydrofurfuryl acrylate, acryloyl morpholine, menthyl acrylate and derivatives of monosaccharides) with classically used 2-ethylhexyl acrylate, mainly. The processes were carried out in the presence of 1-hydroxy-cyclohexyl-phenyl-ketone as a photoinitiator and 1-dodecanethiol as a chain transfer agent [11,12,13,14,15]. Recent research concerns the preparation of PSAs with controlled hydrophilic properties. The authors also used the chain transfer agent (1-dodecanothiol, 0.01 wt %) [16]. In turn, Zhu and at al. investigated the effect of 1,6-hexanediol diacrylate as a crosslinking agent on the performance of PSAs obtained by bulk photopolymerization of *n*-butyl acrylate, 2-hydroxyethyl acrylate and acrylic acid [17].

Telomerization (from Greek telos, meaning “end” and mer meaning “part”) is defined as the reactive process where a molecule YZ (named telogen), reacts onto a polymerizable compound M (named taxogen) to form telomeres of general formula Y(M)_n_Z [18]. The term of telomerization was introduced in 1946 by Peterson and Weber [19,20]. The cleavable bonds of the telogen can be C-H, S-H, P-H, Si-H or C-X, where X = Cl, Br or I [21]. It is noteworthy, that there are some differences between telomerization and polymerization. In telomerization fragments of the initiator mainly induced the cleave bond of the telogen and the number of M units in the final compound is low (*n* < 100), but is not 1. In contrast to polymerization, chain transfer and kinetic chain termination reactions occur much more frequently in telomerization, thus limiting the molecular mass of the reaction products termed telomeres. It should be also noted that telomerization is not a living polymerization but is a non-living reaction to obtained macromolecules [22]. There are known several initiation methods of telomerization reactions: thermal initiation, UV initiation and γ rays [23]. Nevertheless, the most recognized processes are these initiated by thermal initiators. Interestingly, there are only three publications about photoinduced telomerization (photoinitiated reaction between styrene and bromotrichloromethane [24], telomerization of acrylamide in water in the presence of disulfide telogen [25] and telomerization of tetrafluoroethylene initiated by benzoyl peroxide (as photoinitiator and telogen)) [26]. In the literature, relative numerous papers describe the telomerization process of acrylic acid [27,28,29,30,31,32]. In contrast, cotelomerization of acrylic monomers is rarely investigated (cotelomerization of acrylic acid and *n*-octyl acrylate, 2-ethylhexyl acrylate or 2-phenylethyl acrylate in presence of 2-aminoethanethiol hydrochloride [33] and cotelomerization of methyl methacrylate and maleic anhydride with dodecyl mercaptan [34]). In the last decade, the works about telomerization have been directed towards receiving the amphiphilic polymers [35] or block copolymers [36]. The latest scientific reports (2020) concern the preparation of fluorosurfactants [37], preparation of hydrophobic coatings [38] and pioneering studies on telomerization of chitosan and hemicellulose with butadiene in water [39]. Additionally, companies indicate that the telomerization process is particularly practical in industrial terms. In 2007, Dow Chemical on an industrial scale began the production of 1-octene from butadiene by palladium catalyzed telomerization, and since 2019 has been working on the process of telomerization of butadiene with methanol [40].

The bulk photopolymerization processes of acrylic monomers described in the literature are generally completed when the desired prepolymer viscosity is reached. Unfortunately, then the obtained prepolymer is characterized by a high content of unreacted monomers, i.e., 60 wt % or more [10,11,12,13,14]. Potentially, a high monomers content in the prepolymer can negatively affect their stability (pot life), lowers its viscosity (which is not always desirable) and determines the use of additional amounts of crosslinkers and using a top protection layer during UV crosslinking. The work presented here demonstrates a new and environmentally friendly method of obtaining photoreactive PSAs, i.e., a UV-induced cotelomerization process of main monomers (BA and AA). The authors’ intention was to obtain a photoreactive prepolymer (with 4-acrylooxy benzophenone) with the lowest possible content of unreacted monomers. Moreover, the influence of AA concentrations on kinetics of the process and self-adhesive properties of prepared PSAs were studied.

## 2. Materials and Methods

### 2.1. Materials

The following components were used for preparation of BA/AA/ABP cotelomers: n-butyl acrylate (BA), acrylic acid (AA; BASF, Ludwigshafen, Germany) as monomers; 4-acryloylooxy benzophenone (ABP, Chemitec, Scandiccy, Italy) as copolymerizable photoinitiator; ethyl (2, 4, 6-trimethylbenzoyl)-phenyl-phosphinate (Omnirad TPOL, IGM Resins, Waalwijk, The Netherlands) as a radical photoinitiator and tetrabromomethane (TBM; Merck, Warsaw, Poland) as a telogen. The components were applied without purification. A hydroxyl terminated polybutadiene resin Hypro 1200 × 90 HTB (CVC Thermoset Specialties, Emerald Kalama Chemical, Kalama, WA, USA) as a multifunctional monomer of the UV crosslinking process was used.

### 2.2. Synthesis and Characterization of BA/AA/ABP Cotelomers

**Figure 1:**
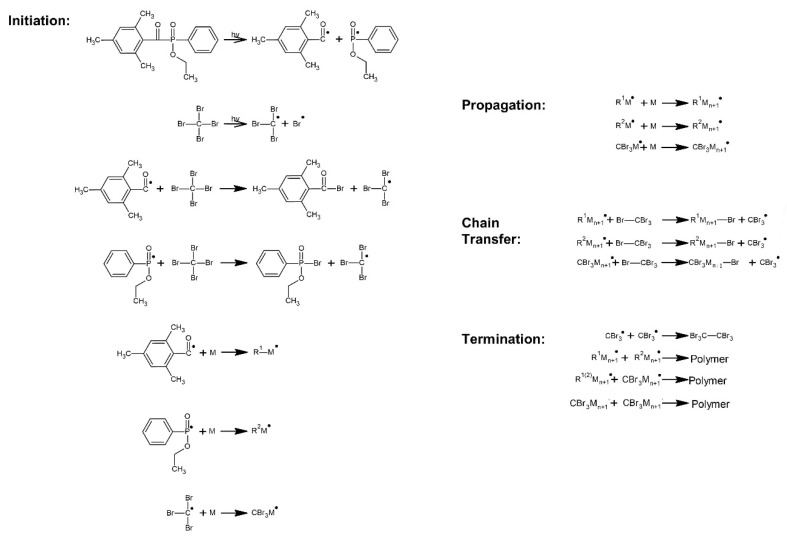
Schematic graph of the UV-induced cotelomerization process.

The cotelomerization process of BA, AA and ABP were initiated using the radical photoinitiator Omnirad TPOL (0.1 phr; per hundred part of the monomers mixture) and tetrabromomethane (2.5 phr) as telogen was used. Mixtures of monomers and samples’ symbols were showed in Table 1. The reaction mechanism was presented in Figure 1.

The cotelomerization processes were realized at 20 °C for 30 min in a glass reactor (250 mL), equipped with a mechanical stirrer and thermocouple, in the presence of argon as inert gas. A mixture of monomers (50 g) was introduced into the reactor and purged with argon for 20 min. The high-intensity UV lamp (UVAHAND 250, Dr. Hönle AG UV Technology, Gräfelting, Germany) as a UV radiation source was used and was placed perpendicularly to the side wall of the reactor. The UV irradiation inside the reactor (15 mW/cm^2^) was controlled with UV-radiometer SL2W (UV-Design, Brachttal, Germany). The reactor was water-cooled (using water in room temperature).

The viscosity of the cotelomers solutions (BAA cotelomers with unreacted monomers, i.e., prepolymers) was measured at 25 °C by means of the DV-II Pro Extra viscometer (spindle #6, 50 rpm; Brookfield, New York, NY, USA). The solid content in cotelomers solutions was determined using Moisture Analyzer MA 50/1.X2.IC.A (Radwag, Radom, Poland). Samples (ca. 2 mg) were heated in aluminum scale pans at the temperature 105 °C for 4 h. Gel permeation chromatography (GPC) was used to determine molecular masses (Mw, Mn) and polydispersity (PDI) of the BAA cotelomers (post-reaction mixtures were dried at 140 °C for 4 h before the test to remove unreacted monomers); the GPC apparatus contained the refractive index detector (Merck Lachrom RI L-7490, Abingdon, UK), pump (Merck Hitachi Liquid Chromatography L-7100, Abingdon, UK) and interface (Merck Hitachi Liquid Chromatography D-7000, Abingdon, UK) and the Shodex Ohpak SB-806 MQ column with Shodex Ohpak SB-G precolumn. The GPC tests were performed using polystyrene standards (Fluka and Polymer Standards Service GmbH, Mainz, Germany) and tetrahydrofurane. The kinetics of the UV-induced cotelomerization process of BA, AA and ABP (composition as in Table 1) were tested using a differential scanning calorimeter with a UV attachment (DSC Q100, TA Instruments, New Castle, DE, USA; UV-light emitter Omnicure S2000; Excelitas Technologies, Malvern, PA, USA) at room temperature (isothermal measurement). Samples (5 mg) were irradiated with UV in the range 320–390 nm with an intensity of 15 mW/cm^2^ in argon atmosphere. All DSC photopolymerization experiments were conducted triplicate. Polymerization rate (Rp, %/s) was calculated according to Equation (1) and conversion of double bonds (p, %)—according to Equation (2) [41].

(1) $$R_{p}=\frac{\left(\frac{\mathrm{dH}}{\mathrm{dt}}\right)}{H_{0}}$$

(2) $$p=\frac{\Delta H_{t}}{\Delta H_{0}}\times 100\%$$

where: dH/dt- heat flow in the polymerization reaction, H_0_—theoretical heat for the complete degree of conversion (for acrylates: ΔH = 78.0 kJ/mol) and ΔH_t_—the reaction heat evolved at time t.

### 2.3. Preparation and Characterization of Obtained Pressure-Sensitive Adhesives (PSAs)

The adhesive compositions for PSAs preparation were compounded using prepolymer (BAA cotelomers with unreacted parts; 90 wt %), HTB crosslinking agent (7.5 wt %) and TPOL photoinitiator (2.5 wt %). The compositions were applied onto polyester foil (50 µm) and UV-irradiated using the medium pressure mercury lamp (UV-ABC; Hönle UV-Technology, Gräfelfing, Germany). The UV doses were 2, 3, 4 or 5 J/cm^2^. The UV exposition was controlled with the radiometer (Dynachem 500; Dynachem Corp., Westville, IL, USA). The basis weight of samples was 60 g/m^2^.

The kinetics of the UV crosslinking process of BAA cotelomers with HTB were tested using a differential scanning calorimeter with a UV attachment (DSC Q100, TA Instruments, USA; UV-light emitter Omnicure S2000; Excelitas Technologies, Malvern, PA, USA) at room temperature. Samples (5 mg) were irradiated with UV in the range 280–390 nm with an intensity of 500 mW/cm^2^. The kinetic curves as the dependence of the heat of reaction on the exposure time have been presented.

Self-adhesive tests (adhesion to a steel, tack and cohesion at 20 °C) were performed at 23 ± 2 °C and 50% ± 5% relative humidity. Adhesion to a steel substrate was tested according to AFERA 4001 and tack acc. to AFERA 4015. The test were carried out with strength machine Zwick Rolell Z010 (Zwick/Roell, Ulm, Germany). The cohesion at 20 °C tests were performed according to FINAT FTM 8. These parameters were evaluated using three samples of each adhesive film.

The spectra of adhesive compositions and PSAs after the UV crosslinking process were obtained using the Fourier transform infrared spectroscopy (Nexus FT-IR, Thermo Nicolet, New Castle, DE, USA). Variation of the absorbance value at 810 cm^−1^ (C=C double bond in acrylates) and absorbance values at 975 cm^−1^ (1,4-cis), 966 cm^−1^ (1,4-trans) and 911 cm^−1^ (1,2-vinyl) of double bonds in HTB were monitored. 

## 3. Results

### 3.1. Properties of BAA Cotelomers

The results of the dynamic viscosity test and solid content for obtained BAA cotelomers solutions with different content of acrylic acid (5; 2.5; 5; 7.5 or 10 wt %) are shown in Table 2.

**Figure 2:**
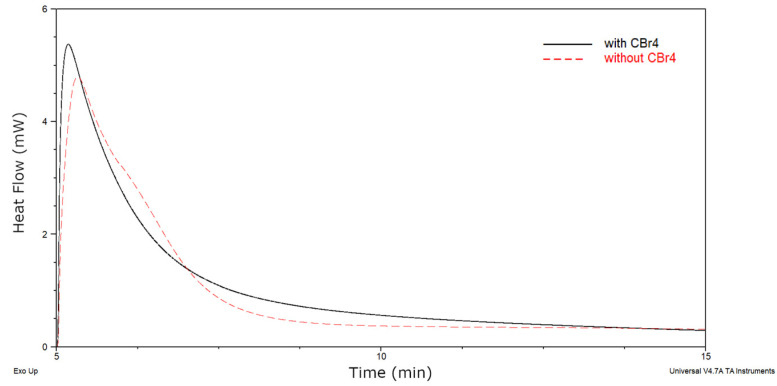
UV-induced cotelomerization curves for monomers compositions (BA—94 wt %, AA—5 wt %, ABP-1 wt % and TPOL-0.1 phr) with CBr_4_ (2.5 phr) and without CBr_4_ (I_o_ = 15 mW/cm^2^ and 320–390 nm).

As can been seen, the viscosity and solid content of cotelomers solutions decreased significantly as the acrylic acid content increased (η = 47.6 Pa∙s and SC = 91.4% for sample without AA and η = 9.1 Pa∙s and SC = 70.3% for BAA cotelomer with 10 wt % of AA). Interestingly, the molecular weights measurements exhibited very similar values of molecular weights (M_n_ ca. 12,200–14,100 g/mol and M_w_ ca. 18,600–21,700 g/mol) regardless of the AA content. The obtained values are much lower than in the case of bulk photopolymerization without the use of a classical chain transfer agent [17]. Additionally, the PDI values amounted to ca. 1.52–1.55 a.u. (slightly lower value of PDI was found for the sample without acid, i.e., 1.51 a.u.). That means that obtained cotelomers characterized low polydispersity index as in the case of polymers obtained by the ATRP method and near unimodal molecular weight distribution (1.1–1.2) [42]. It is known that molecular weight distribution of polymers affects the mechanical properties [43]. Preparation of polymers with such a low PDI index and low molecular weights was possible due to the use of a chain transfer agent (CBr_4_). Additionally CBr_4_ acts as telogen in the process. Due to this, the obtained products can be called cotelomers. Interestingly, the photo-DSC measurements revealed that CBr_4_ also can act as photoinitiator. Barson and coauthors showed that UV-induced telomerization of styrene in the presence of bromotrichloromethane without the photoinitiator taking place [24]. They proved that CCl_3_Br acts both as a photoinitiator and as a transfer agent. We tested two systems: without CBr_4_ and with CBr_4_ (both contained a photoinitiator TPOL). The photo-DSC thermogram was shown in Figure 2. It is known that heat flow rate is proportional with the polymerization rate [44]. The system containing CBr_4_ reached a higher value of heat flow than the system without CBr_4_, hence the conclusion that a sample with a telogen polymerized faster. On this basis, it was found that the CBr_4_ molecule also acted as a photoinitiator (the same as CCl_3_Br in the Barson’s work). The observed increase in the reaction rate was most likely due to the generation of additional radicals from the telogen molecule.

**Figure 3:**
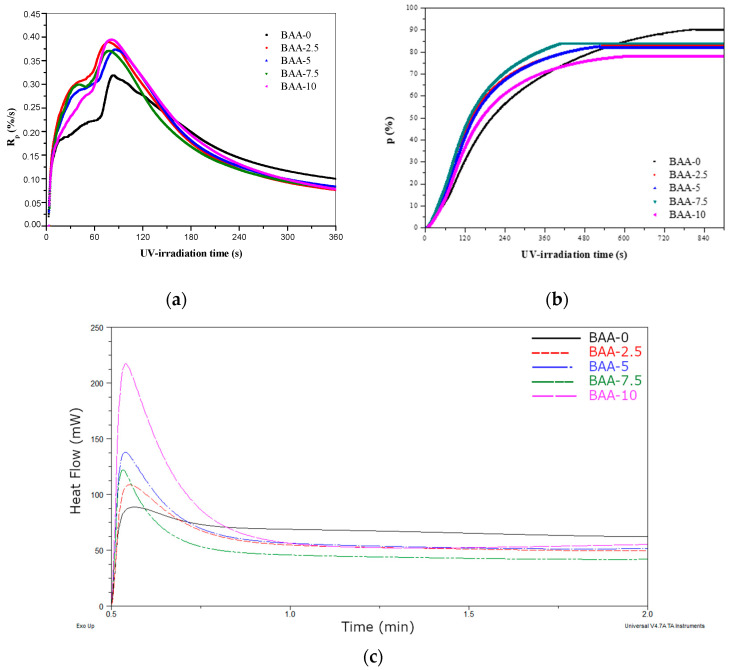
Cotelomerization rate curves (**a**), conversion spectra (**b**) and photo-DSC curves (**c**) of UV-induced cotelomerization of BA and ABP and various amount of AA in the presence of TPOL photoinitiator and CBr_4_ (I_0_ = 15 mW/cm^2^; 320–390 nm).

The heat flow for a sample with CBr_4_ was higher than for samples without CBr_4_. Additionally, photo-DSC measurements revealed that the UV-induced cotelomerization process was faster in the presence of acrylic acid. In Figure 3a the kinetics curves were shown. The polymerization rate (R_p_) values were higher for systems with AA (the highest values was noticed in case of 10 wt % of AA). It is also characteristic that the maximum reaction speed (maximum peak on the curves) was reached after ca. 90 s of UV irradiation. Moreover, a second peak on the curve is discernible (most visible in the case of sample BAA-7.5), which may confirm a double way of initiating a reaction (by radicals generated from photoinitiator and telogen photolysis). Regarding the conversion of acrylate groups (p, Figure 3b), it should be noted that the highest final conversion was achieved by the sample without AA (90%) but after the longest UV irradiation time (ca. 840 s). This is due to the lower polymerization rate (R_p_) of this system (Figure 3a). Samples with 2.5 and 5 wt % of AA exhibited a very similar profile of conversion curves and the final conversion value (82% and 83%, respectively). The sample BAA-7.5 also achieved high final conversion (84%), but in shorter UV irradiation time (ca. 360 s). The lowest value of final conversion was noticed to the system with 10 wt % of AA (75%). In the case of a sample without AA (BAA-0), it can be said that we are dealing with a linear photopolymerization of a difunctional monomer (ignoring the ABP influence, which is only 1 wt % in the system). It is known from the literature that such systems are characterized by high conversion values [45].

In contrast, systems with AA behaved like multicomponent (multifunctional) systems, which were characterized by high reactivity (R_p_) at the beginning of the reaction, there was a phenomenon of autoacceleration and consequently the conversion was lower. The slowdown of the reaction (visible after 180 s of irradiation for systems with AA, Figure 3a) the carboxyl group could induce (the possibility of creating interchain hydrogen bonds in the systems, which makes access to double bonds difficult and inhibition of polymerization). It is worth noting that the conversion of acrylate groups’ results (Figure 3b) was quite similar to the solids content in the systems after the cotelomerization process in the reactor. The differences in the achieved results were caused by the process conditions. Although the UV dose and the irradiation time were identical (in the glass reactor and during the photo-DSC test in aluminum pans), the reaction in the reactor was carried out by mechanical mixing of the components. 

### 3.2. Properties of PSAs Based on BAA Cotelomers

**Figure 4:**
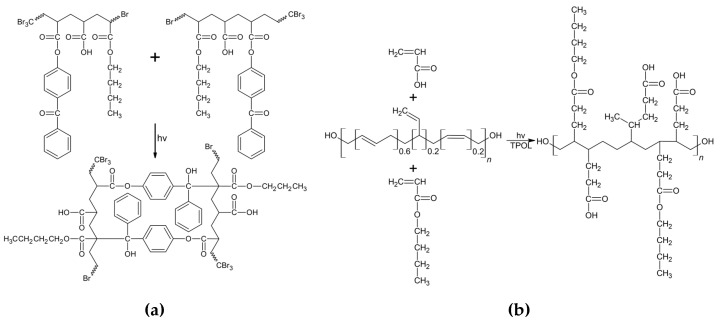
Photocrosslinking in pressure-sensitive adhesives (PSAs): (**a**) via copolymerizable photoinitiator ABP (hydrogen abstractor) and (**b**) via HTB and unreacted monomers.

Based on the prepared BAA cotelomers solutions (with unreacted monomers), the hydroxyl terminated polybutadiene resin Hypro 1200 × 90 HTB and additional dose of photoinitiator TPOL, the PSAs films were prepared. Figure 4 shows the possible cross-linking reactions that occurred when the adhesive film was exposed to the UV lamp (equipped with UV-C, UV-B and UV-A lamps). It is known from the literature that the ABP photoinitiator belongs to the type II photoinitiators and its action was based on the abstraction of hydrogen from the coinitiator molecule. For pressure-sensitive adhesives, the coinitiators were considered to be the tertiary carbon atoms of the monomer molecules. 

**Figure 5:**
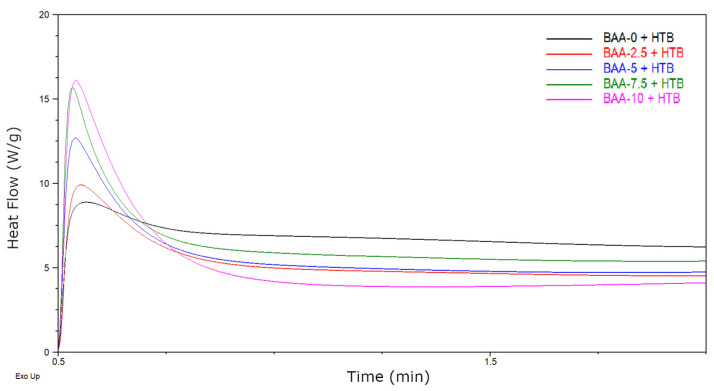
Photo-DSC curves of adhesive compositions (I_0_ = 500 mW/cm^2^; 230–390 nm).

This process takes place under the influence of UV-C radiation [46,47]. An illustrative mechanism of the reaction is shown in Figure 4a. The second type of reaction that takes place during the exposure of adhesive films is cross-linking with HTB and unreacted monomers (BA and AA). The reaction is shown in Figure 4b. In this way a semi-interpenetrating polymer network (semi-IPN) is to create. The course of these processes was examined by photo-DSC. The samples (BAA cotelomers solutions, HTB and TPOL) were irradiated in the range of the UV-A,B,C region with a UV dose of 500 mW/cm^2^. The photo-DSC curves are shown in the Figure 5. As would be expected, the reaction rate increased with the acrylic acid content, which in fact is due to the higher content of unreacted monomers in systems with higher AA content.

**Figure 6:**
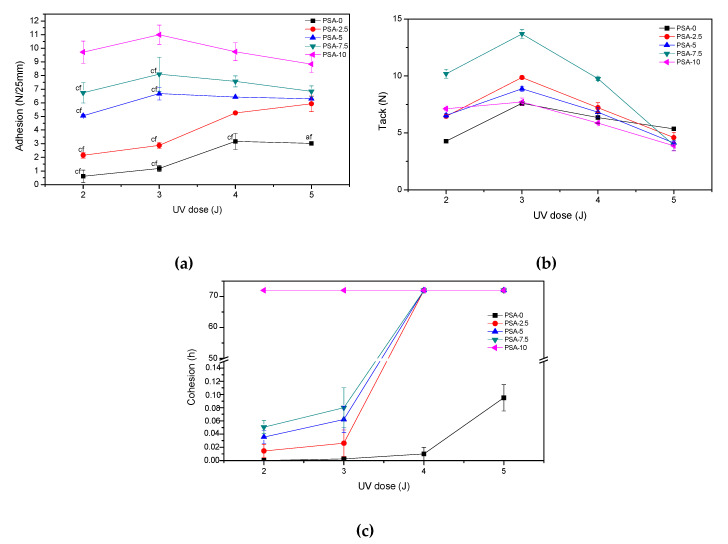
Self-adhesive features of PSAs based on BAA cotelomers: (**a**) adhesion to steel (cf-cohesive failure), (**b**) tack and (**c**) cohesion.

The self-adhesive properties of PSAs film based on BAA cotelomers, i.e., adhesion to steel substrate, tack and cohesion at 20 °C, tested according to the UV dose used in the step of the crosslinking process (2; 3; 4 or 5 J/cm^2^) were presented in Figure 6. As can be seen in Figure 6a, adhesion values for all PSAs films UV crosslinking with doses 2 or 3 J/cm^2^ increased, regardless of the AA content. In contrast, for samples PSA-0 and PSA-2.5 the adhesion values increased with an increase of UV dose, while for PSA with more content of AA a decrease in adhesion values was noted. It is related to the higher cross-linking density of the samples PSA-5; PSA-7.5 and PSA-10, which contained more acrylic acid and more unreacted monomers in adhesive composition before the UV crosslinking process. It is known from the literature that an increase in the cross-linking density of adhesives reduces their adhesion [48]. In the case of samples with a high acid content (PSA-10), no damage was observed, regardless of the UV dose used. The conducted tests also proved that the adhesion of PSAs increased significantly with the increase of acrylic acid content. For PSA-10 the adhesion values reached 9–11.5 N/25 mm and for reference sample PSA-0 only 0.5–3 N/25 mm. The increase in adhesion was the result of an increase in the content of carboxyl groups in adhesives and the formation of hydrogen bonds with the steel surface. In turn, the tack values decrease with an increase in the UV dose (from 3 to 5 J/cm^2^), regardless of the AA content. In the case of this test, the interaction between surface and PSA film was less important (the contact time with the test surface was only a few seconds, while in the adhesion test the adhesive film adhered to the surface for 20 min before tearing off). In the case of samples with a high acid content (PSA-10), no damage was observed, regardless of the UV dose used. In turn, the tack values decreased with an increase in the UV dose (from 3 to 5 J/cm^2^), regardless of the AA content. In the case of this test, the interaction between surface and PSA film was less important (the contact time with the test surface was only a few seconds, while in the adhesion test the adhesive film adhered to the surface for 20 min before tearing off).

**Figure 7:**
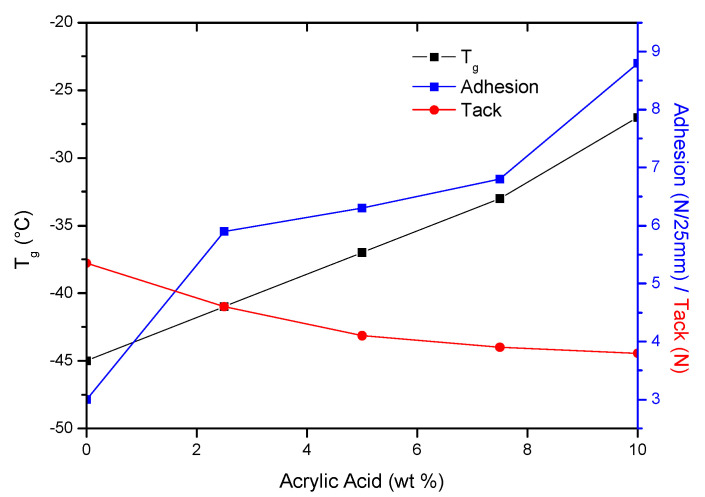
Adhesion, tack and T_g_ of PSAs as a function of AA content.

More important is the stiffness of the adhesive film, which determines its ability to wet/stick to the substrate. The sample PSA-7.5 was distinguished among all samples. Probably a good balance between crosslink density and polar carboxyl groups content in PSA-7.5 gave it higher tack. It is also known that adhesion and tack of PSAs depend on glass transition temperature of the base polymer [49]. Glass transition temperatures were determined for samples after the crosslinking process with the UV dose of 4 J/cm^2^ (PSAs were free from adhesive failures). The results are shown in Figure 7 as the dependence of the adhesion, tack and glass transition temperature on the acrylic acid content.

**Figure 8:**
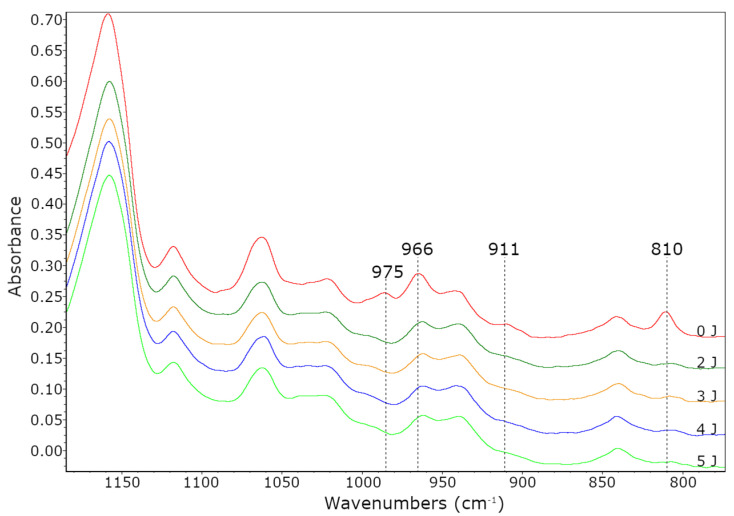
FTIR spectra of PSA-7.5 after the UV crosslinking process.

The T_g_ values increased with increasing AA content (from −45 to −27 °C, for 0 wt % and 10 wt % of AA, respectively). It is known that the glass transition temperature of poly(acrylic acid) is about 100 °C. Hence, its higher content in samples caused an increase in their T_g_ values. As the T_g_ values of PSAs increased, the adhesion increased and their tack decreased. The reduction in stickiness is caused by limitations in the mobility of the polymer chains at a higher T_g_, thereby worse wetting the surface. The third feature of PSAs adhesives is their cohesion. The results of performed tests are shown in Figure 6c. As can be seen, the highest desired result (72 h) was achieved in the case of sample PSA-10, regardless of the applied UV dose. This is most likely due to two factors. Firstly, PSA-10 contained the most carboxyl groups, hence the most hydrogen bonds in the system. Secondly, PSA-10 had the most unreacted monomers before UV crosslinking, hence the densest polymer network was formed at the cocrosslinking stage with HTB (a large proportion of crosslinking polymerization over linear polymerization). For these reasons, the PSA-10 sample had the highest cohesion. In case of sample PSA-7.5 the cohesion values after crosslinking using 4 or 5 J/cm^2^ of UV dose were also excellent. However, the presence of unreacted monomers had a greater negative effect than HTB, since the latter was only 7.5 wt % in the samples and unreacted acrylate monomers from ca. 9–30 wt %. The characteristic peaks (810 cm^−1^; 911 cm^−1^ and 966 cm^−1^ and 975 cm^−1^) of PSAs crosslinked with different UV dose were monitored by FTIR spectroscopy. An exemplary FTIR spectrum (for PSA-7.5) was shown in Figure 8. This adhesive was selected for the tests due to the fact that it had the highest tack of all prepared PSAs, high adhesion and the desired cohesion after irradiation with a UV dose of 4 J/cm^2^. With the increasing of UV dose, the intensity of peaks 810 cm^−1^, 911 cm^−1^ and 975 cm^−1^ disappeared (especially after using a high UV dose, i.e., 4 and 5 J/cm^2^). That means, that all unreacted acrylate monomers and vinyl double bonds in HTB reacted completely. The pendant double bonds (vinyl-) and 1,4-cis double bond in HTB were readily available and therefore participated readily in crosslinking. Instead, the trans- double bonds were not fully reacted and were still present in the final product (but in a low concentration). Probably, this is due to the difficult accessibility of this type of bonds and steric hindrances in the HTB chain.

## 4. Conclusions

In this paper, the UV-induced cotelomerization process as a new and environmentally friendly method of obtaining PSAs was presented. The effect of acrylic acid content on kinetics of the reaction and selected features of obtained prepolymers and self-adhesive properties of PSAs were studied. We stated that as the acrylic acid content increased:

- The rate of the reaction (R*p*) increased,
- The conversion of the monomers, solid content in prepolymers and its viscosity values decreased,
- The adhesion and cohesion values increased and tack values decreased; only in PSA-10 with the highest AA content, the highest values of adhesion and cohesion were obtained (and adhesive film were free from failures), regardless of the applied UV dose.

Interestingly, that AA content had practically no influence on the molecular weights of the obtained cotelomers and their polydispersity. The optimal AA content in PSAs based on BA/AA/ABP cotelomers was found to be 7.5 wt %—the highest tack (10 N), adhesion (7.5 N/25 mm) and cohesion (72 h) values after UV crosslinking with 4 J/cm^2^ of UV dose were reached. Besides, it was found to achieve the highest monomer conversion (84%) in the shortest time (360 s).

## Figures and Tables

**Table 1:** Compositions of regents for the UV-induced cotelomerization process.

Cotelomers Acronym	Monomers (wt %)			Telogen (phr)	Photoinitiator (phr)
	BA	AA	ABP		
BAA-0	99	0	1	2.5	0.1
BAA-2.5	96.5	2.5	1	2.5	0.1
BAA-5	94	5	1	2.5	0.1
BAA-7.5	91.5	7.5	1	2.5	0.1
BAA-10	89	10	1	2.5	0.1

**Table 2:** Dynamic viscosity, solid content and molecular weights of BAA cotelomers.

Cotelomers Acronym	η (Pa∙s)	SC (%)	M_n_ (g/mol)	M_w_ (g/mol)	PDI
BAA-0	47.6	91.4	13,470	20,290	1.51
BAA-2.5	29.0	87.2	12,210	18,630	1.53
BAA-5	19.6	82.6	12,870	19,590	1.52
BAA-7.5	14.5	76.3	14,115	21,710	1.54
BAA-10	9.1	70.3	13,810	21,415	1.55
